# Development of an education campaign to reduce delays in pre-hospital response to stroke

**DOI:** 10.1186/s12873-017-0130-9

**Published:** 2017-06-24

**Authors:** Caterina Caminiti, Peter Schulz, Barbara Marcomini, Elisa Iezzi, Silvia Riva, Umberto Scoditti, Andrea Zini, Giovanni Malferrari, Maria Luisa Zedde, Donata Guidetti, Enrico Montanari, Mario Baratti, Licia Denti, On behalf of the Educazione e Ritardo di Ospedalizzazione (E.R.O.I) study group, Paola Castellini, Carla Zanferrari, Annalisa Tanzi, Francesca Diodati, Silvia Olivato, Filippo Barbi, Guido Bigliardi, Maria Luisa Dell’Acqua, Laura Vandelli, Francesca Rosafio, Roberta Pentore, Livi Picchetto, Daniela Monaco, Eva Perticaroli, Ilaria Iafelice, Paolo Imovilli, Luca Vaghi, Angelica Guareschi

**Affiliations:** 1 Research and Innovation Unit, University Hospital of Parma, Via Gramsci 14, 43126 Parma, Italy; 2 Institute of Communication and Health, Università della Svizzera italiana, Via Buffi 6, CH 6900 Lugano, Switzerland; 3 Department of Oncology and Hematology, University of Milan, Via Festa del Perdono 7, 20122 Milano, Italy; 4 Stroke Care Program, Neurology Unit, University Hospital of Parma, Via Gramsci 14, 43126 Parma, Italy; 5 Stroke Unit, Nuovo Ospedale Civile S Agostino Estense, Via Pietro Giardini 1355, 41126 Baggiovara, Modena Italy; 6 Department of Neuromotor Physiol, Stroke Unit, Neurology Unit, Arcispedale Santa Maria Nuova IRCCS, Viale Umberto I 50, 42123 Reggio Emilia, Italy; 7 Department of Neurology, Guglielmo da Saliceto Hospital, Via Taverna 49, 29100 Piacenza, Italy; 8 Neurology Unit, Vaio Fidenza Hospital, Via Don Tincati 5, 43036 Fidenza, Parma Italy; 9 Division of Neurology, B Ramazzini Hospital, Via Guido Molinari 2, 41012 Carpi, Modena Italy; 10 Clinical Geriatrics Unit, University Hospital of Parma, Via Gramsci 14, 43126 Parma, Italy

**Keywords:** Stroke, Public campaign, Pre-hospital delay, Media, Cartoon, Intervention mapping

## Abstract

**Background:**

Systematic reviews call for well-designed trials with clearly described intervention components to support the effectiveness of educational campaigns to reduce patient delay in stroke presentation. We herein describe the systematic development process of a campaign aimed to increase stroke awareness and preparedness.

**Methods:**

Campaign development followed Intervention Mapping (IM), a theory- and evidence-based tool, and was articulated in two phases: needs assessment and intervention development. In phase 1, two cross-sectional surveys were performed, one aiming to measure stroke awareness in the target population and the other to analyze the behavioral determinants of prehospital delay. In phase 2, a matrix of proximal program objectives was developed, theory-based intervention methods and practical strategies were selected and program components and materials produced.

**Results:**

In phase 1, the survey on 202 citizens highlighted underestimation of symptom severity, as in only 44% of stroke situations respondents would choose to call the emergency service (EMS). In the survey on 393 consecutive patients, 55% presented over 2 hours after symptom onset; major determinants were deciding to call the general practitioner first and the reaction of the first person the patient called. In phase 2, adult individuals were identified as the target of the intervention, both as potential “patients” and witnesses of stroke. The low educational level found in the patient survey called for a narrative approach in cartoon form. The family setting was chosen for the message because 42% of patients who presented within 2 hours had been advised by a family member to call EMS. To act on people’s tendency to view stroke as an untreatable disease, it was decided to avoid fear-arousal appeals and use a positive message providing instructions and hope. Focus groups were used to test educational products and identify the most suitable sites for message dissemination.

**Conclusions:**

The IM approach allowed to develop a stroke campaign integrating theories, scientific evidence and information collected from the target population, and enabled to provide clear explanations for the reasons behind key decisions during the intervention development process.

**Trial registration:**

NCT01881152. Retrospectively registered June 7 2013

**Electronic supplementary material:**

The online version of this article (doi:10.1186/s12873-017-0130-9) contains supplementary material, which is available to authorized users.

## Background

In the past few decades, public education campaigns have been widely used to influence health behaviour, but they have not always shown positive results. Different hindrances, including inadequate funding, an increasingly cluttered media environment, use of poorly researched messages, the power of social norms and the drive of addiction, can make campaigns unsuccessful, or even counterproductive [1].

Education campaigns aimed to reduce delays in prehospital response to life-threatening disorders, such as acute myocardial infarction and acute stroke, are no exception to this [2]. Systematic reviews of interventions aimed at improving emergency response to stroke show that, while they are capable of improving the public’s knowledge and understanding of symptoms, as well as the need for emergency care, their effects on relevant clinical outcomes such as hospital arrival times and thrombolysis treatment rates have been most often disappointing [3, 4].

It must be pointed out, however, that most published studies show a number of methodological limitations, such as lack of a control arm and insufficient power, which limit the robustness of their conclusions. Also, most studies did not report any theoretical base of the intervention, and for the majority there was no description of intervention development and no mention of any modelling and exploratory or pilot work to test the processes, necessary elements recommended for the design of complex interventions [5] and for the reporting of quality improvement initiatives [6]. The aforementioned systematic reviews therefore emphasize the need for well-designed research trials with clearly described intervention components to provide evidence for the effectiveness of interventions [3, 4].

In the past few years, research has been conducted to investigate determinants of decision-making that lead to action at stroke onset, since it has become evident that this information is crucial for the development of effective education interventions to reduce time to hospital arrival [7, 8]. A systematic review [7] including 182 studies found that more severe strokes and strokes with symptoms regarded as serious were among the factors associated with shorter time delay, but better knowledge about the most frequent stroke symptoms was not. The authors concluded that there was a discrepancy between knowledge of stroke symptoms and the reaction to the occurrence of one or more of these symptoms. Recently, the concept of “stroke preparedness” has been introduced, meaning the ability of lay individuals to recognize stroke symptoms and take immediate action to seek emergency treatment, which should be the focus of any educational intervention to reduce pre-hospital delay [9].

A number of approaches to public education about stroke symptoms have been suggested (10), such as: culturally appropriate descriptions of the signs and symptoms of stroke; campaigns also targeted at audiences other than the potential stroke patient, e.g. children; emphasis on the existence of therapies that can improve outcomes and minimize disability; emphasis on the fact that symptoms of stroke, even when they do not seem severe or dramatic, are the manifestation of a serious disease process requiring immediate medical intervention.

Furthermore, a preliminary assessment of the local barriers and facilitators to immediately seeking emergency treatment is considered a key element in designing educational interventions with some potential for success, because understanding behaviors includes making predictions about why people behave the way they do [10, 11].

Based on these considerations, our group developed an education campaign to increase stroke awareness (recognition of symptoms and making sense of their seriousness) and preparedness, targeting the general population, building on a strong theoretical base, and on the analysis of survey data for local context analysis. To this end, we followed a systematic process of intervention development guided by the Intervention Mapping framework [12], which provides a system for the integration of theory, empirical findings from the literature, and information collected from the target population to identify the most appropriate education strategy, also ensuring reproducibility of the methodology.

This paper describes in detail the planning process of the public campaign, the effectiveness of which will be tested in a large randomized trial with stepped-wedge design (trial registration NCT01881152).

The research questions this work intended to answer where the following:

1. What is the perceived severity of stroke symptoms in the general population?
2. What actions are performed by patients at the time of symptom onset? How do behaviors of patients who arrive promptly at the hospital differ from those who arrive late?
3. Which elements should the message contain to remove obstacles and to be more effective?

## Methods

### Setting

This study was carried out in four geographically contiguous provinces of Emilia Romagna (a region in Northern Italy), with 1,975,763 inhabitants, of whom 423,382 (21.4%) are aged over 65 years. The four provinces constitute the Wide Area North Emilia (AVEN), one of the three areas established by the region to rationalize spending and optimize the efficiency of health services. The area comprises 22 community hospitals, 2 teaching hospitals and 1 scientific research institute. Each province is served by at least one stroke unit. The number of patients discharged with a diagnosis of first-ever acute stroke (ICD-9-CM in primary diagnosis = 433.x1, 434.x1) is 3050/year [13].

The work presented here is part of a multicenter, randomized trial funded by the Emilia-Romagna Region in the framework of the “Programma di ricerca Regione-Università 2010-2012” grant, aiming to assess the effectiveness of a population-based information-education campaign, in terms of reduction of delay in hospital admissions for acute stroke. The project, promoted by the volunteer association ALICe (Associazione per la Lotta all’Ictus Cerebrale – Association for Fighting Cerebral Stroke) [14], was approved in 2012 by the Ethics Committees of all participating centers. All involved subjects gave written informed consent to participate. The duration of the entire study is three years, and this first part was conducted between February and August 2013.

### Study design

The educational campaign was developed following IM, a tool that guides researchers in selecting target behaviors, specifying intervention goals, choosing intervention strategies and planning the implementation. The development process in this study was articulated in two phases: needs assessment and intervention development. In the first phase, two surveys were performed, a population and an in-hospital survey.

In the second phase, the following three steps were applied [12, 15]:

- Developing matrices of change
- Selecting theory-based methods and strategies
- Designing and organizing the program

### Phase 1 (needs assessment)

According to IM, before beginning to actually plan an intervention, the planner assesses the health problem, its related behavior and environmental conditions, and their associated determinants for the at-risk populations. To this end, we conducted a population and an in-hospital survey, aiming to measure stroke awareness and preparedness in the target population, and to analyze the behavioral determinants of prehospital delay, defined as patient admission later than 2 hours after symptom onset. The 2-hour cut-off was chosen because it is the maximum pre-hospital delay that allows for eligible patients to receive thrombolysis within 3 hours. We did not extend the time window for eligibility because, at the time this study was designed, thrombolysis three hours or more after stroke onset stroke onset was not recommended for patients over 80 years old [16], who usually represent a substantial proportion of the stroke population.

#### Population survey

The survey was conducted with cross-sectional design on citizens over 18 years of age, living in the AVEN Provinces. To increase sample representativeness, in each province subjects were recruited at three different sites, one for each area (urban, rural, mountain). These sites included fitness centers, recreation centers for senior citizens, theaters, shopping malls, churches, football stadiums. To facilitate recruitment, the survey was conducted in the framework of events organized by ALICe aimed to sensitize the public on stroke and its prevention, e.g. distribution of information material, video broadcasting, and debates on healthy lifestyle. The questionnaires were administered at the recruiting sites by a neurologist and a representative of ALICe, who approached citizens and invited them to take part in the survey, without applying any exclusion criteria. Participants, who joined the survey voluntarily, filled in the questionnaire and returned in on site.

Stroke awareness was assessed using the Stroke Action Test (STAT) questionnaire [17], cross-culturally adapted to the Italian context [18], following the methodology described by Beaton [19] and performing pretest evaluation on 30 volunteers to improve clarity and comprehension.

The STAT was used since it can not only assess the respondent’s theoretical knowledge of stroke warning signs, but also his/her ability to connect symptoms with appropriate actions. The STAT is self-administered, and includes 28 closed-ended questions, each concerning a hypothetical scenario: 21 stroke symptoms representing all 5 groups of warning signs commonly used to convey stroke symptoms in clinical and public health settings and among advocacy organizations (sudden confusion, trouble speaking or understanding speech; sudden numbness or weakness of face, arm or leg, especially on one side of the body; sudden trouble seeing in one or Both eyes; sudden trouble walking, dizziness, loss of balance or coordination; sudden severe headache with no known cause), as well as 7 non-stroke symptoms. For each scenario, the respondent is asked to select 1 of 4 options: call EMS, call doctor, wait 1 hour, or wait 1 day. The primary outcome measure was the STAT score, which is the average number of questions (only considering the 21 items relating to stroke symptoms) for which the respondents would call EMS. Our secondary outcome measure was the percentage of respondents who correctly indicated they would call EMS for more than 10 stroke symptoms (>50% of 21) [20, 21]. The instrument also contains one question investigating which local health care information sources are most widely used, and gathers some demographic data (gender, area of residence, age class) to investigate the association of these variables with responses.

#### In-hospital stroke survey

This survey, conducted with cross-sectional design, aimed to detect the factors that influence behavior, through the analysis of the actions that are put in place at the time of symptom onset by patients (or their caregivers) who arrive at hospital in a timely manner (≤2 hours of symptom onset), compared to those who arrive later. This enabled us to define intervention objectives and analyze health problems. The questionnaire used for data collection was selected from the literature. At the time the protocol was designed, we identified two instruments used to investigate reasons for pre-hospital delay [22, 23]. We judged the tool by Carroll et al [23] to be unpractical for our study, as it consisted in open-ended questions administered to patients in a standardized, structured interview, and analysis of responses would be challenging. We thus chose the questionnaire by Hsia et al [22], which comprises ten questions regarding initial impression, first action, transport mode and delay in hospital arrival. Patients who arrived later were also asked to select from a pre-specified list of factors perceived as barriers to an early admission.

The questionnaire was administered within 72 hours of hospital admission to all patients consecutively hospitalized at the participating centers for acute stroke. Only first ever strokes were enrolled, to prevent the confounding effect of a previous stroke experience, which per se might elicit a more prompt reaction to symptom onset. Subjects with uncertain symptom onset time were also excluded. Questionnaires were administered during structured interviews performed by neurologists who had attended 2 half-day training sessions aimed to ensure uniformity of behavior and good quality of collected data. If the patient could not be interviewed because of aphasia, severe dysarthria, or altered mental status, then a relative or friend (proxy) who was with the patient at the time of presentation was interviewed.

For all patients, data were collected concerning: age, sex, education, type of stroke according to the Oxfordshire Community Stroke Project (OCSP) classification [24], vascular risk factors and comorbidities. Furthermore, for patients who were able to respond without the need of a proxy, the level of health literacy was estimated by a validated screening instrument, the Brief Health Literacy Screen (BHLS), a verbally administered, three-question survey [25, 26]. The test was properly modified to make it appropriate to the local context. The respondents were asked to answer three questions: 1. How often do you have someone help you read hospital materials? 2. How often do you have problems learning about your medical condition because of difficulty understanding written information? 3. How often do you have problems learning about your medical condition because of difficulty understanding oral information from medical personnel? The latter question has been changed from the original (How confident are you filling out medical forms by yourself?), because in our context patients are not frequently asked to fill in medical forms. Patient responses were recorded in the electronic medical record on a 5-point scale ranging from 0 = never to 4 = always, with higher scores indicating lower health literacy. According to available evidence [26, 27], the response cutoff for optimal sensitivity and specificity for low health literacy corresponds to a score of 2 (i.e., “some of the time”) on each item of the scale.

### Phase 2 (intervention development)

In this phase, the results of needs assessment were taken into account to select the most appropriate theoretical foundation of the campaign, to identify its objectives, and for each of these, the psychological constructs involved and the changes in people behavior that could be expected from the intervention.

To plan the intervention, a Steering Committee (SC) was formed, comprising the Principal Investigator, three referring clinicians for the participating centers, one communication expert, one methodologist, one psychologist, one representative of ALICe, one EMS physician, one journalist, and the representative of the communication agency in charge of campaign organization.

To provide indications for campaign development, the SC met six times over a period of 3 months, and, considering two theoretical models, applied the aforementioned steps:

#### Developing matrices of change

The SC specified three levels of objectives: desired behaviours (behavioural outcomes), a breakdown of these behaviours (performance objectives) and modifiable objectives formed by crossing the determinants with performance objectives.

#### Selecting theory-based methods and strategies

In this step, a list of potential intervention methods that are matched to the objectives stated in the proximal program matrix was generated. They were taken from a number of theoretical or empirical techniques used to influence people’s behavior available in the literature [28]. In particular, modelling, narration, skill development and persuasive communication were taken into account and discussed in relation with the findings from the two surveys. The most appropriate strategies, i.e. the practical ways of delivering the intervention method, were then selected among the ones usually employed for educational interventions that address large numbers of heterogeneous people, i.e. the so-called mass strategies [28].

#### Designing and organizing the program

In this final step, the SC organized the strategies into a deliverable program taking into account target groups and settings, and producing and pretesting the materials. Separate strategies were integrated into one coherent, multilevel campaign, and decisions made on the program’s structure, its theme, the sequence of strategies and communication vehicles. In this phase, the SC closely collaborated with the creative team of the communication Agency in charge of campaign implementation.

To test the educational products and to identify the most suitable sites for message dissemination, five focus groups were conducted, between March 2013 and October 2013. The first two focus groups recruited participants from the Province that was going to be exposed to the campaign first, according to the randomized sequence, while the others included participants from each of the other three communities involved in the campaign. Each group comprised a convenience sample of participants, heterogeneous according to age, gender and education level, included at least one individual with a history of stroke and, according to recommendations for qualitative studies, shared a common knowledge about stroke and the local resources available to patients and caregivers [29, 30].

Focus groups should involve a sufficient number of people to ensure heterogeneity of the provided information, yet they should not be too large because this may discourage participants from sharing their feelings, views, opinions, and experiences.

A single moderator with expertise with focus groups (SR) guided all group discussions according to a pre-planned agenda of questions. Different combined data were collected during the interview including notes taken by the moderator and items recalled by the moderator and assistant moderator. The discussion was a balance between obtaining answers to the questions, and hearing from each participant in their own words.

Each session consisted of five parts: signing of informed consent, warm-up discussion about participants’ experience with stroke, presentation of educational product prototypes, exchange of views on the product prototypes, and identification of the public places and public events deemed more suitable for campaign dissemination. In particular, participants were asked to examine the prototypes of the educational products and give explicit suggestions on various aspects: the reliability of the message, its specificity to the problem of stroke, the use of the narrative method and of the cartoon, the layout , clarity, and the efficacy of the key messages. To quantify evaluation, a 10 point Lickert scale was used to rate each participant’s approval of each aspect.

The unit of analysis was the group. Data were coded and emergent themes highlighted, to provide interesting information about the main topics that were discussed [31].

### Data and statistical analysis

To verify sample representativeness in the community survey, demographic characteristics of respondents were compared with demographics official data (produced by the Italian National Institute of Statistics – ISTAT) [32]. In order to assess the possible associations with questionnaire responses, the characteristics of respondents who correctly indicated they would call EMS for more than 10 stroke symptoms (>50% of 21) and of the remaining respondents were compared (prevalent outcome event). As for the hospital survey, demographic characteristics, medical conditions and the behavioural response to symptom onset were compared between patients with timely arrival at hospital (≤2 hours after symptom onset) and those with late arrival (prevalent outcome event). Because both surveys use a cross-sectional design, and because factors for which association with the outcome is to be measured are stable (age, sex, behaviors, etc.) or in any case precede the event [33, 34], the association was measured using the Prevalence Ratio (PR) estimates and corresponding confidence interval (CI 95%). To determine the degree of association and statistical significance, the log-linear binomial model was used. This regression model was chosen because it is the best performer in the cross-sectional study design [35]. Multivariate analysis was performed, considering delayed hospital presentation as the dependent variable, and as independent covariates the demographic, clinical and behavioral variables which were shown to be statistically significant in the univariate analysis (*p* < 0. 20).

All analyses were performed with SAS version 8.2. The PR was estimated with the Proc Genmod command with logarithmic link function.

### Sample size

The number of subjects to include in the community survey was determined on the basis of the findings of two studies [17, 20], which report mean scores on the 21 items containing stroke symptoms between 25% and 35%. Assuming a power of 0.80 and an alpha error of 0.05, overall 173 questionnaires were deemed necessary. To ensure representation of subgroups with specific characteristics, proportionate stratification (for geographical area, sex, age class) was used.

For the in-hospital survey, sample size considerations were more informal. According to data collected at the University Hospital of Parma on 1847 patients included in the hospital’s Stroke Care Pathway from 2003 to 2011, only 512 (27.7%) subjects with suspected stroke presented at hospital within 2 hours of symptom onset [36]. Based on this finding, to ensure sufficient precision of estimates, and considering the limited time allowed for the survey, we calculated that about 400 patients with first episode of stroke or TIA should be enrolled. Prerequisites for patient inclusion were written informed consent, and ability to trace back the circumstances present at symptom onset (either the patient was conscious, or bystanders were present who could be interviewed at the hospital).

## Results

### Phase 1 (Needs assessment)

#### Population survey

Between February and March 2013, 202 questionnaires were collected at the 12 community-based sites. As shown in Table 1, the sample’s demographic characteristics coincide with those of the target population, according to official statistics [32]. Specifically, 52% of healthy volunteers resided in a rural area, 55% was female, the prevailing class age was 40-64 years (48%) and only 14% had university education.

**Table 1:** Demographic characteristics of respondents compared with those of the general population

	Sample	Populations^a^
Demographic characteristics	(*n* = 202)	(*n* = 3.848.550)
Area		
urban	70 (34% )	1.500.935 (39%)
rural	105 (52%)	1.770.333 (46%)
mountain	27 (14%)	577.283 (15%)
Gender	*(1 missing value)*	
Male	91 (45%)	1.853.259 (48%)
Female	110 (55%)	1.995.291 (52%)
Age		
20-39	56 (28%)	1.011.386 (28%)
40-64	97 (48%)	1.588.385 (44%)
>65	49 (24%)	1.007.452 (28%)
Education	*(4 missing values)*	
Primary school (1-5 years) or no education	31 (16%)	781.033 (20%)
Secondary school (6-8 years)	43 (22%)	1.128.140 (29%)
High school (9-13 years)	95 (48%)	1.421.088 (37%)
University or graduate education	28 (14%)	518.289 (14%)

^a^Source: Emilia-Romagna Resident (Year 2013 - dati.istat.it)

Readiness to respond to stroke, measured with the STAT questionnaire, is shown in Table 2. The mean overall STAT score (based on all 28 items) was 52.5% (SD 16.6). The mean score on the 21 items containing stroke symptoms was 44% (SD 21.8). This means that on average, participants chose to call “118”, the EMS number in Italy, in 44.3% of the situations with stroke symptoms. They chose to call their doctor for 26.0% of the stroke symptom situations, wait 1 hour in 22.3%, and wait 1 day in 6.7%.

**Table 2:** Answers to items containing stroke symptoms (*N* = 21 items*202 participants)

Selecting	% answer (*n* = 4242)
Call “118” immediately	1880 (44%)
Call doctor's office immediately	1102 (26%)
wait 1 hour and then decide	945 (22%)
wait 1 day and then decide	283 (7%)
missing	32 (1%)

**Fig. 1:**
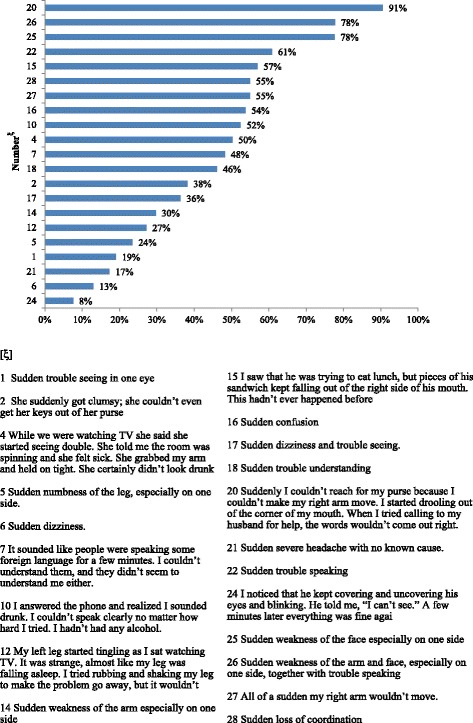
Responses to STAT. STAT scenarios ordered by percentage of correct responses

As shown in Table 3, only 82 out of 202 respondents (41%) indicated they would call EMS for at least 50% of 21 items, showing that most underestimated the seriousness of stroke symptoms. The respondents who performed best (recognized more than 50% of stroke scenarios as urgent) were older and lived more often in the rural area (Table 3), while no difference was found in terms of education. The expressed intention to call EMS was indicated by over 75% of respondents for both scenarios concerning myocardial infarction warning signs (items 13 and 23), and for 3 of 21 items related to stroke (Fig. 1): arm weakness that presented together with trouble speaking (91%), sudden weakness of the arm and face together with trouble speaking (78%), and sudden weakness of the face especially on one side (78%).

**Table 3:** Sociodemographic characteristics of subjects who would call “118” for more than 10 stroke symptoms (>50% of 21) compared with those of the remaining respondents

	>50%items (%) *N* = 82	≤50%items (%) *N* = 120	Prevalence Ratio	CI (95%)	Pr > Chisq
Gender *(1 missing value)*					
Male	35(43%)	56 (47%)	1		
Female	47 (57%)	63 (53%)	0,93	0.74-1.17	0,539
Age Class					
20-39	15 (18%)	41 (34%)	1		
40-64	45 (55%)	52 (43%)	0,73	0.56-0.93	0,011
> = 65	22 (27%)	27 (23%)	0,75	0.56-1.01	0,062
Education *(4 missing value)*					
Primary school (1-5 years) or no education	14 (18%)	17 (14%)	1		
Secondary school (6-8 years)	22 (28%)	21 (18%)	0,90	0.58-1.38	0,631
High school (9-13 years)	36 (45%)	59(50%)	1,14	0.81-1.61	0,441
University and over	8 (10%)	21 (18%)	0,90	0.90-1.93	0,161
Area					
Urban	22 (27%)	48 (40%)	1,25	1.00-1.56	0,052
Rural	50 (61%)	55 (46%)	0,79	0.65-0.99	0,041
Mountain	10 (12%)	17 (14%)	1,07	0.78-1.47	0,657

The stroke symptoms for which the lowest number of participants would call EMS were a transient visual loss (8%), sudden dizziness (13%), sudden severe headache (17%) and sudden trouble seeing in one eye (19%). In these cases, over 2/3 of respondents chose “call doctor’s office” or “wait 1 hour”.

**Fig. 2:**
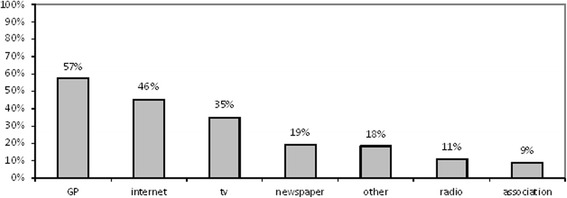
Information sources. Local sources and channels of health information most commonly used by respondents

As for the most used local source of health care information (Fig. 2), 57% of participants indicated their general practitioner (GP), and only 19% the newspapers.

#### In-hospital survey

**Fig. 3:**
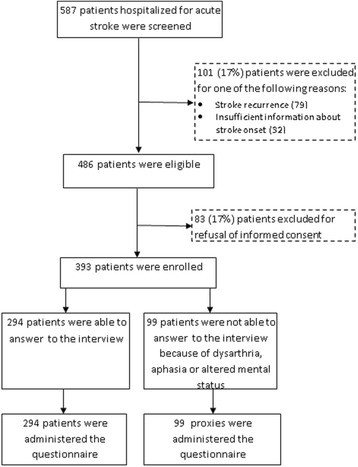
Flow diagram

Between March and June 2013, 587 patients with suspected stroke were admitted to the hospitals of the 4 participating provinces (Fig. 3). Of these, 101 were not included either because the event was a recurrence or no information was available concerning the time of stroke onset. Of the 486 eligible patients, 83 did not sign the informed consent. Thus, the final sample was of 393 patients.

The median time from symptom onset/awareness to presentation at the hospital in the sample was 2 hours and 22 minutes (IQR 1 hour and 33 min–8 hours and 7 minutes). 218 patients (55%) presented more than 2 hours after stroke onset/symptom awareness, defined as delayed access. 245 (62%) arrived by ambulance, making up 81% (142/175) of those who arrived at hospital within 2 hours, and 47% (103/218) of those with delay (Table 4).

**Table 4:** Mode of arrival at hospital: comparison between those who arrive promptly and late

	≤2 hours *N* = 175	>2 hours *N* = 218	Total *N* = 393	Prevalence Ratio	CI 95%	Pr > Chisq
Ambulance/ “118”	142 (81%)	103 (47%)	245 (62%)	0.54	0.46-0.64	<0.001
Personal/ Relative’s/Friend’s car	32 (18%)	105 (48%)	137 (35%)	1.74	1.47-2.05	<0.001
Other	1 (1%)	10 (5%)	11 (3%)	0.90	0.22-3.61	0.883

Table 5 shows the patients’ sociodemographic and clinical characteristics in relation to the delay. Median age was 75 (IQR 65-83) years, 199 (51%) were women and 215 (55%) had primary school education. Over 90% of the sample had at least 1 stroke risk factor. The two groups exhibit similar demographic characteristics, whereas differences were observed regarding: living alone (PR = 1.28 IC95% 1.07-1.53, *p* = 0.008), presence of hypertension (PR = 0.82 IC95% 0.68-0.97, *p* = 0.025) and the occurrence of ischemic stroke type TACI (PR = 0.56 IC95% 0.36-0.87, *p* = 0.09) or LACI (PR = 1.48 IC95% 1.24-1.76, *p* < 0.001). Health literacy was estimated for 294 patients. Over 50% of our sample exhibited low literacy, although this did not appear to be directly associated with delay.

**Table 5:** Patient sociodemographic and medical characteristics according to time of arrival at hospital

	≤2 hours *n* = 175	>2 hours *n* = 218	Total *n* = 393	Prevalence Ratio	CI 95%	Pr > Chisq
Age						
≥65 years	133 (76%)	155 (71%)	288 (73%)	0.90	0.74-1.08	0.260
Median (IQR)	76 (66-84)	74 (64-83)	75 (65-83)			
Gender						
Female	86 (49%)	113 (52%)	199 (51%)	1.05	0.88-1.25	0.596
Education *(3 missing values)*						
Primary school (1-5 years) or no education	99 (58%)	116 (53%)	215 (55%)	1		
Secondary school (6-8 years)	40 (23%)	54 (25%)	94 (24%)	1.08	0.87-1.34	0.483
High school (9-13 years)	24 (14%)	35 (16%)	59 (15%)	1.11	0.87-1.42	0.385
University or graduate education	9 (5%)	13 (6%)	22 (6%)	1.11	0.76-1.61	0.578
Lives alone						
Yes	30 (17%)	60 (28%)	90 (23%)	1.28	1.07-1.53	0.008
Risk factors						
Prior TIA	25 (14%)	29 (13%)	54 (14%)	0.96	0.74-1.26	0.782
Hypertension	135 (77%)	147 (67%)	282 (72%)	0.82	0.68-0.97	0.025
Diabetes	29 (17%)	42 (19%)	71 (18%)	1.08	0.87-1.35	0.476
Smoker	31 (18%)	41 (19%)	72 (18%)	1.03	0.83-1.29	0.778
Hyperlipidemia	48 (27%)	56 (26%)	104 (26%)	0.96	0.78-1.18	0.701
Atrial fibrillation	40 (23%)	42 (19%)	82 (21%)	0.91	0.72-1.14	0.401
Carotid artery disease	21 (12%)	21 (10%)	42 (11%)	0.89	0.65-1.22	0.474
Ischemic heart disease	27 (15%)	38 (17%)	65 (17%)	0.90	0.45-1.81	0.767
Peripheral vascular disease	4 (2%)	4 (2%)	8 (2%)	1.07	0.85-1.34	0.585
None	12 (7%)	25 (11%)	37 (9%)	1.25	0.98-1.59	0.076
Diagnosis						
Transient ischemic attack (TIA)	24 (14%)	34 (16%)	58 (15%)	1.07	0.84-1.35	0.590
Hemorrhagic stroke	20 (11%)	19 (9%)	39 (10%)	0.87	0.62-1.21	0.402
Ischemic stroke:	123 (70%)	149 (68%)	272 (69%)	0.96	0.80-1.16	0.676
*TACI (Total Anterior Circulation Stroke Infarct)*	*29 (17%)*	*14 (6%)*	*43 (11%)*	*0.56*	*0.36-0.87*	*0.009*
*PACI (Partial Anterior Circulation Stroke Infarct)*	*54 (31%)*	*60 (28%)*	*114 (29%)*	*0.93*	*0.76-1.14*	*0.478*
*POCI (Posterior Circulation Stroke Infarct)*	*27 (15%)*	*32 (15%)*	*59 (15%)*	*0.97*	*0.76-1.25*	*0.838*
*LACI (lacunar Stroke)*	*13 (7%)*	*43 (20%)*	*56 (14%)*	*1.48*	*1.24-1.76*	*<.0001*
Other	8 (5%)	16 (7%)	24 (6%)	1.22	0.90-1.64	0.194

**Fig. 4:**
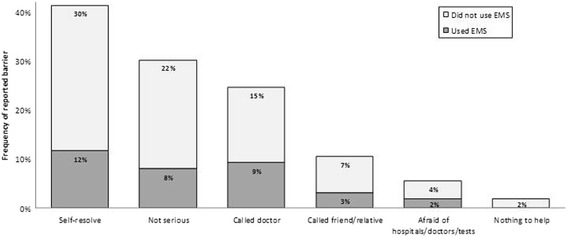
Patient-reported barriers. Frequencies of barriers reported by patients who arrived at hospital after 3 hours, subdivided into patients who did and did not use EMS

Figure 4 depicts frequencies of barriers reported by the 162/393 patients (41%) who arrived at hospital after 3 hours, subdivided into patients who did and did not use EMS. Although the definition of delayed arrival in this study is after 2 hours, reported barriers refer to a >3-hour delay, because only this information is investigated by the tool of Hsia et al [22]. Clearly, most patients who did not use EMS underestimated symptom severity, believing either that they would self-resolve (30%), or that they were not serious (22%).

Patient and/or caregiver response to symptom onset was specifically explored by three questions, concerning two of the major themes recognized as able to influence the decision to seek help at the time of stroke: making sense of symptoms, and the presence and influence of another person (Table 6). Only 23% (89/393) of patients/proxies reported that they realized symptoms might be related to a stroke/TIA. Making sense of symptoms was associated with pre-hospital delay, since attributing symptoms to other diseases was positively associated with a delayed admission to hospital (PR = 1.23 IC95% 1.08-1.53, *p* = 0.004).

**Table 6:** Actions taken at the time of symptom onset by patients (or their caregivers) who arrive early at the hospital (≤2 hours of symptom onset) vs those who arrive later

When you developed the symptoms that brought you to the hospital, what did you FIRST think was wrong?	≤2 hours *N* = 175	>2 hours *N* = 218	Total *N* = 393	Prevalence Ratio	CI 95%	Pr > Chisq
Stroke/TIA	47 (27%)	42 (19%)	89 (23%)	0.82	0.64-1.04	0.094
Other	46 (26%)	86 (39%)	132 (34%)	1.23	1.08-1.53	0.004
I have not thought about anything	82 (47%)	90 (41%)	172 (44%)	0.90	075-1.08	0.273
Whom did you first call or speak with after your symptoms started?						
“118”	35 (20%)	9 (4%)	44 (11%)	0.34	0.19-0.62	<0.001
General Practice (GP)	11 (6%)	62 (28%)	73 (19%)	1.74	1.50-2.02	<0.001
Relative/Friend	115 (66%)	134 (61%)	249 (63%)	0.92	0.77-1.10	0.379
Other	14 (8%)	13 (6%)	27 (7%)	0.86	0.57-1.28	0.461
What was the reaction of the person you first called or spoke with after your symptoms started?						
Encouraged me to call “118”	74 (42%)	45 (21%)	126 (32%)	0.57	0.44-0.73	<0.001
Encouraged me to call my GP	3 (2%)	25 (11%)	28 (7%)	1.69	1.44-1.98	<0.001
Encouraged me to go to the hospital	8 (5%)	54 (25%)	63 (16%)	1.72	1.49-2.00	<0.001
Drove me to the hospital	19 (11%)	24 (11%)	43 (11%)	1.01	0.76-1.34	0.961
Recommended that I wait to see if my symptoms improved	2 (1%)	11 (5%)	13 (13%)	1.55	1.21-1.99	0.001
Other	69 (39%)	59 (27%)	88 (22%)	0.82	0.67-1.02	0.072

Only 11% of participants first called “118”, whereas most first referred to a relative or a friend. Calling the GP was associated with a delayed admission (PR = 1.74 IC95% 1.50-2.02, *p* < 0.001).

The third question (Table 6) concerned the behavior of the person to whom the patient first referred. Only 31% advised to call EMS, which was associated with a low rate of delayed admission (PR = 0.57 IC95% 0.44-0.73, *p* < 0.001), while other types of advice, such as encouraging the patients to call the GP, to go to the hospital, or to wait and see, were associated with a higher probability of delayed admission.

In the multivariate analysis (Table 7), factors that were shown to significantly increase delay were deciding to call the GP first (pr = 1.15 CI95% 1.03-1.27, p = 0.009) and the reaction of the first person the patient called (pr = 1.12 CI95%1.03-1.23, *p* = 0.013).

**Table 7:** Log-Binomial Model

	Prevalence Ratio	CI 95%	Pr > Chisq
Lives alone (Yes = 1)	1.06	0.95-1.17	0.263
Risk factor Hypertension (Yes = 1)	0.96	0.88-1.05	0.387
Diagnosis Ischemic Score LACI (lacunar Stroke) (Yes = 1)	1.06	0.96-1.17	0.238
I first think (Other = 1)	1.03	0.96-1.12	0.401
I first call or speak ( GP = 1)	1.15	1.03-1.27	0.009
The reaction of the person I first called (Encouraged me to call “118” = 0)	1.12	1.03-1.23	0.013

### Phase 2 (Intervention development)

The theoretical foundation guiding our intervention had to be focused on individual capacity (as decision to take action after stroke onset pertains to the patient himself or his/her relative), had to address the response to an acute disease, and should consider as the target population not only individuals at risk of stroke, but any person who might witness stroke onset. Many health-behavior theories focused on individual capacity exist [28], mostly employed in the field of prevention or management of chronic, rather than acute, diseases. Among them, the SC selected the General Model of Total Patient Delay, initially proposed by Safer et al. [37] and modified by Andersen et al [38], because it offers a framework to study and improve understanding of health-seeking behavior. Andersen’s model describes the delay as comprised of four stages (appraisal, illness, behavioral and scheduling delay intervals), each governed by a conceptually distinct set of decisional and appraisal processes, from the first occurrence of an unexplained symptom to the time when the individual appears before a physician. The first stage (appraisal delay) describes the time a person takes to evaluate somatic information and decide whether it is indicative of illness. The second stage (illness delay) describes the time taken from deciding one is ill to deciding the illness requires, or will be ameliorated by professional care. The third stage (behavioral delay) describes the time between a person deciding an illness requires medical attention and deciding to act on this decision. The fourth stage (‘scheduling delay’) describes the time between deciding to act on the decision to seek help and the actual access to any medical evaluation. The description of patient behavior as a sequence of stages was deemed as a useful guide to properly analyze pre-hospital delay in the in-hospital survey.

The SC also considered the Common Sense Model (CSM) of self regulation [39]. The CSM explains how individuals respond to and manage health threats. It has been prevalently applied to chronic illnesses, but the SC considered that some aspects of the model could be applied to the acute setting of stroke. Notably, the key construct within the CSM is the idea of illness representations or ‘lay’ beliefs about illness, which enables people to make sense of symptoms and guides any coping actions. According to the model, coping decisions will differ as a function of the meaning individuals assign to their symptoms (i.e. their illness representation), and this interpretive process will reflect their past illness experience, societal expectations, information from friends, media, and medical practitioners. Furthermore, the model identifies two parallel processes of illness representation, cognitive and emotional, and organizes the cognitive representation into five dimensions (identity, timeline, control, consequences and causes), which all motivate to seek care.

As for the General Model of total patient delay, the in-hospital survey clearly confirmed that the delay in patient response to stroke can be represented according to a sequence of stages, each one related with the delay and influenced by specific determinants. Furthermore, the underestimation of the symptoms, found by both surveys, and its contribution to the delay, confirmed the importance of stroke representation as determinant of patient/proxy’s behavior, especially in terms of cognitive processes. Two domains of cognitive representation of stroke were considered particularly important by the SC for the development of the educational message: identity (suggested by the low level of stroke symptom knowledge and recognition found in both surveys), and control (suggested by the high proportion of patients referring to someone else as first reaction to stroke).

As for the evidence from the literature, some of the major stroke awareness campaigns have been evaluated in terms of educational tools and outcomes employed, notably the NINDS Know Stroke campaign [40], the American Heart Association (AHA) Power to End Stroke campaign [41], the Stroke Heroes Act FAST campaign of the Massachusetts Department of public health [42], Stroke Warning Information for Faster Treatment (SWIFT) [43, 44] and the UK Act- FAST campaign [45, 46].

#### Developing the matrix of program objectives

We started from the assumption that low stroke recognition, not immediately referring to the EMS or scarce knowledge about treatment opportunities in the first few hours (notably, thrombolysis) are determinants of pre-hospital delay. Such assumption is supported by evidence from the literature [7, 10, 47] and has been partly confirmed in our context. Importantly, the in-hospital survey, which enabled us to describe patient and proxy help seeking behavior, according to the General Model of total patient delay [37, 38] confirmed in our context a low rate of stroke symptom recognition (appraisal delay), a low rate of first referrals to the EMS (illness and behavioral delays) and the significant contribution of these factors to the overall pre-hospital delay (Tables 6 and 7). The low level of stroke awareness in the general population confirmed the need to address people’s illness representation to improve their response to stroke onset, according to the CSM of self regulation [39].

As for the knowledge about thrombolysis, it was not explored by the two surveys, therefore no data on the local context were available. It is noteworthy, however, that, differently from other reports [11], in our context a low proportion of patients (7%) mentioned the belief that nothing can be done to treat a stroke as a barrier to calling the EMS. Therefore, the committee considered the evidence from the literature [11, 47] as sufficient to support the final decision to include the knowledge of potential for stroke treatment among the performance objectives of the intervention. For each performance objective, the corresponding behavioral outcomes are defined and represented in Table 8.

**Table 8:** Matrix of program objectives according to Intervention Mapping recommendations

Performance objectives	Modifiable determinants			Behavioral outcomes
	Knowledge	Self-efficacy	Outcome expectation	
Recognizing symptoms as related to stroke	Being able to recognize the most frequent stroke symptoms	Feeling confident about recognizing the symptoms		Prompt reaction to stroke as un urgency by the patient or any witness of stroke onset (perception, interpretation and appraisal)
Realizing symptoms seriousness	Knowing that stroke is a serious disease			Seeking immediately medical professional care (decision making)
Being aware that timely arrival will give access to treatments that can lead to complete recovery	Knowing the treatment opportunity for stroke, notably thrombolysis		Trusting in the possibility of complete recovery with specific treatments	Referring immediately to the hospital (decision making)
Calling EMS immediately	Knowing that the first thing to do is to call EMS	Feeling confident about being able to do the right thing by oneself	Trusting in EMS as a mean to arrive timely to the hospital	Use EMS (ambulance) to go to the Hospital (decision making)

As for the modifiable determinants, 3 main psychological constructs were identified: knowledge, self-efficacy and outcome expectation. The committee agreed that evidence from the two surveys was sufficient to confirm in our context an influence of knowledge and self-efficacy. Notably, the association of recognition of symptoms by patients or proxies as related to stroke with an earlier access indicates knowledge as a determinant of delay. Furthermore, the high proportion of patients referring first to others (relatives, friends and GP) instead of immediately calling an ambulance was considered as an indicator of low self-efficacy. Outcome expectation was included among the determinants mainly according to literature evidence, as already mentioned [10, 11, 47].

#### Selecting theory-based methods and strategies

Adult individuals, independent of age, sex, education and risk level, were identified as target of the intervention. In fact, findings from our patient survey, in line with data from the literature [48] and with theoretical assumptions, clearly showed that in most cases (89%) patients turned to someone else to decide what to do in response to symptom onset and that any witnesses of stroke onset can play a central role in the decision-making process. The intervention should therefore target all individuals, not only high-risk subjects.

The wide target audience of the intervention, as well as the low educational level and low health literacy of most respondents of the patient survey, called for a simplified mode of message delivery, such as narrative communication, defined as “a representation of connected events and characters that has an identifiable structure, is bounded in space and time, and contains implicit or explicit messages about the topic being addressed” [49, 50].

The cartoon form was chosen by the committee, because according to available evidence, pictures closely linked to written or spoken text can markedly increase attention to and recall of health education information compared to text alone. This is particularly true for old people and persons with low health literacy [51, 52]. In fact, it has been suggested that visual images in cartoons, combined with the text, activate different processing systems in the brain which have been shown to improve understanding [53] and increase recall of medical information [54]. Finally, the cartoon form has been shown to be very efficient as it allows to communicate a story in a simple way, using colors and words [51].

Among the several possible ways of organization of the message content (appeals), the committee decided to avoid the threatening or fear-arousing method, which might lead to people denying and rejecting the message. This decision was taken based on the shared understanding that most people view stroke as a life-threatening disease typical of the elderly with no potential for recovery, and thus usually prefer to ignore the problem. As a matter of fact, a high proportion of patients or caregivers in the in-hospital survey (47%) declared that they “did not think of anything” about the symptoms. The message thus had to be organized in such a way to act on people’s tendency to disregard the problem as a threatening issue. Furthermore, since our message is “positive” (the aim is to promote beneficial behaviors), the fear-arousal appeal would be less appropriate than a persuasive approach focused on arousing people interest, limiting harm perception. Overall, the message was organized to provide instructions and hope, an approach already used in other studies [11, 42]. For this reason, the committee decided that the message should include the concept of treatability of stroke with rapid action, as well as promote preparedness and self-efficacy, including as key messages “Spare time, gain life”, “118. Call me” and “You can make the difference”.

#### Designing and organizing the program

A range of characters were featured: the members of a family, including the grandfather as the typical stroke patient, the grandmother, who performs the appropriate actions, the young nephew and his mother, and a super-hero representing the Emergency Service (Additional file 1). The choice of the family setting was deemed appropriate in the light of data obtained from the patient survey; 77% of respondents did not live alone, and 73% was over 65 years old. Also, the family proved to be the most important resource of promotion of correct behavior: 43% of individuals who arrived at hospital within 2 hours had been advised by a family member to call EMS.

The story we chose to tell was about a patient whose relative (his wife), at the onset of stroke symptoms, immediately calls the ambulance, and thus arrives timely at hospital, which leads to a complete recovery. The story represents the stages of an appropriate response to stroke, according to the General Model of Patient Delay, and places emphasis on a correct representation of stroke as a serious disease with potential for full recovery. Overall it addresses the four performance objectives of the previously developed matrix.

The previously designed characters were embedded into a comic strip, a poster and an animation video. The storyboard of the comic strip was designed as a sequence of frames, starting with the picture of the ambulance running to the hospital, and ending with the family at the hospital gathered around the patient who has completely recovered. The representation of the old patient’s wife, an old lady herself, as a person able to do the right thing (calling 118) and confident about her ability to act without referring to others, was intended to promote self efficacy. In a distinct frame, one character (the patient’s wife) explains the symptoms. This way we addressed in the comic strip the three determinants we intended to modify (knowledge, self efficacy and outcome expectation). The super-hero representing the 118 service is shown both in the comic strip and in the poster. The animation video displays, in a sequence of distinct frames, the most common stroke symptoms.

The following educational products were created: a brochure depicting the comic strip; a poster depicting the super-hero; an animation video for closed circuits; an animation video clip for TV broadcasting.

The overall campaign strategy, in terms of communication tools and message delivery channels, was designed based primarily on the available evidence. Two reports were taken into account [10, 55], which, using sufficiently rigorous designs (one cluster randomized and one quasi- experimental trial), demonstrated the efficacy on relevant endpoints, such as hospitalization delay and frequency of thrombolysis use, of two types of intervention: an educational letter indicating stroke symptoms and emphasizing the importance of calling the emergency medical services, mailed to the households [55] and a multilevel strategy, developed according to a strict methodology and largely employing mass media (television and radio [10]).

The results of our surveys highlighted a discrepancy between the sources of health information people recognized as reliable (population survey) and the sources that actually gave information about stroke in the local context (in-hospital survey). Overall, it appears that information about stroke available through the channels viewed as the most reliable are scarce, so that people mostly rely on their personal relationships. Such a discrepancy can be only partly explained by the age and education differences between the two samples (stroke patients were much older and less educated than the participants in the population survey.

The committee viewed the finding about the high proportion of respondents who mentioned their interpersonal relations as the source of knowledge about stroke as in support of high grade of social cohesion and community orientation that is typical of our population. This finding suggests that public events should be included among the delivery channels of the campaign, as they offer the opportunity to spread the educational message.

Finally, economic and methodological constraints, the latter in relation to the experimental design selected for intervention efficacy assessment (cluster randomized stepped wedge trial), were discussed. These limited the use of mass media, most of all television, because of the high costs and the high risk for “contamination” across clusters [56].

The final decision was in favor of a multilevel campaign, employing the following as delivery channels:

- Mail delivery of the brochures to the households of the participating provinces
- Display of brochures and posters in several public places (hospitals, malls, pharmacies and headquarters of volunteer organizations). Because of the high degree of reliability of the GP as the preferred source of health information, community health centers were also identified as sites for educational product display.
- Broadcast of the closed-circuit animation video in public places, such as waiting rooms of Emergency Departments.
- Broadcast of the animation video clip on the local television stations.
- Putting up exhibit booths for distribution of educational products in the framework of public events, such as street and town fairs, and weekly markets.

For the five focus groups, a total of 35 participants (13 men, 22 women) aged 44-78 years were recruited (6-8 participants for each group). Each session lasted 60 minutes. Focus groups were audiotaped, transcribed, and analyzed. The moderator used a matrix sheet to facilitate information collection. According to responses to the open-ended questions, the key messages were easily perceived by most participants, who, after the first examination of the products, were able to clearly recall the description of symptoms , the need to call 118 and the good outcome of the story told in the cartoon (patient full recovery) as the most salient components of the message content. The use of the cartoon was the object of extensive discussion. Some participants perceived the representation of stroke in cartoon form to be too optimistic and, as such, unrealistic; also, some felt it may not be suitable for elderly people. There was general agreement on the efficacy of depicting 118 as a super-hero.

Credibility, specificity, the use of the narrative method and the efficacy of the key messages were all rated as “high” by most participants. The layout of the brochure was instead rated as “low”, because of issues with the graphics and the storyboard. Lack of clarity, mainly due to the graphics used rather than to message representation, was also object of debate.

Based on these findings, suggestions to improve the cartoon strip were provided to the graphic designer, who revised the prototypes. No substantial change was made to the text, while some minor improvements to the cartoon’s layout and enlargement of text font were suggested, to improve readability. As for the selection of public places and events for campaign dissemination, most of those proposed by the Communication Agency were confirmed by the participants.

The overall campaign strategy was planned to ensure that all intervention components, in terms of structural, temporal, and topographic characteristics, were standardized enough to be homogeneous across the four provinces. Standardization was also considered a pre-requisite to perform monitoring throughout campaign implementation [57].

The mail delivery of the brochures, the display of posters and brochures in public places and the closed-circuit animation video broadcast is the first phase of the educational campaign, which will be followed by monthly “reinforcements” by participation in public events and the periodic restocking of the educational products in public places.

According to the stepped-wedge design, which implies that the campaign will be launched sequentially in the four provinces with 3 month intervals, campaign duration will differ in the four communities, lasting for a maximum of twelve months in the first province and a minimum of three months in the last province exposed to the intervention.

Broadcasting of the animation video clip on the local TV stations will be limited to the final month of the campaign, when all four clusters are exposed to the intervention, avoiding the risk that television from an already exposed province overlaps into a neighboring province not yet exposed to the intervention.

970,000 brochures depicting the comic strip will be mailed to households; additional 30,000 brochures and 400 posters depicting the Super-Hero will be displayed in public places. The closed-circuit animation video will be broadcast for at least 30 days during the campaign in each community. Educational products will be distributed during public meetings on a monthly basis, so the number of meetings in each province will depend on campaign duration.

## Discussion

In this study, we described in detail the development of a public education campaign aimed at reducing pre-hospital delay of stroke patients, based on the findings of two surveys on the target population, which were integrated with theoretical assumptions and literature evidence, following a standardized methodology, i.e. the Intervention Mapping approach.

In this way, we intended to address one of the limitations emphasized by published systematic reviews on the effectiveness of education interventions to increase stroke awareness, which show that most public campaigns are generally designed without evidence of prior context analysis and theoretically grounded development of the interventions [3, 4]. In fact, a description of the process of intervention development was made available only for some of the major stroke preparedness campaigns so far published: the TLL Temple Foundation Stroke [10], the SWIFT (Stroke Warning Information and Faster Treatment) Study [43, 44] and the ASPIRE (Acute Stroke Program of Interventions Addressing Racial and Ethnic Disparities) Study [11]. These interventions were all designed using acute stroke patient or population data collected by different methods, such as focus group session, population or patient surveys and key informant interviews.

In our study, we aimed to give a detailed description of the process of campaign development, both to allow reproducibility, as required in the SQUIRE reporting guidelines [6], and to enable comparison with other stroke campaigns. Unlike the above mentioned reports, focus groups were only used to test the educational products and to identify the most suitable sites for product distribution in the four provinces. In this regard, we judged that the focus group method, pertaining to qualitative research, would be very appropriate for campaign prototype testing before definite approval, as it allows to obtain in-depth information on few cases and to explore a wide range of unstructured or semi-structured response options. We instead considered the focus group method to be unsatisfactory for Phase 1 context analysis, because of the need for more objective, statistically valid and generalizable information that can only be provided by quantitative methods, such as surveys.

First of all, the two surveys yielded useful information for needs assessment, which was the necessary premise to the development of the matrix of program objectives. Overall, they confirmed the high prevalence of patients admitted to the hospital later than 2 hours after symptom onset and the contribution of each stage of the process of help-seeking to the total delay. Most of all, we identified making sense of symptoms (attributing symptoms to stroke and realizing their severity) and prompt referral to the EMS as significant components of the delay. Also, according to the analysis on patients’ perceived barriers to early hospital referral, the most frequently mentioned reason for delay was patients’ initial belief that their symptoms were not serious and/or did not require treatment. These findings are in line with those reported by Hsia et al. [22], who indicate inability to recognize stroke signs and symptoms as a potential explanation for the observed discrepancy between behavioral intent of citizens and actual behavior of hospitalized patients. The need for increasing stroke awareness was also supported by the fact that in the population survey, 60% of respondents indicated a correct response (call EMS) for less than 50% of the items.

Based on these findings, the educational message was designed to address two main issues: stroke symptoms description and the need to immediately call EMS, a choice also supported by Bray et al [58], who found that the FAST stroke awareness campaign was most effective when the message “Call an ambulance” was added.

As for symptom description, the population survey revealed that some clinical hypothetical scenarios, such as those representing motor impairment, were more often perceived as serious, compared with other scenarios, such as any symptoms related to visual impairment, suggesting that a wide range of stroke symptoms must be covered in the message, emphasizing that even the manifestation of a single one of them should not be overlooked. Therefore, the five groups of warning signs commonly used to describe stroke symptoms were covered in the message. It must be pointed out, however, that stroke signs and symptoms were not the focus of our message, since the literature shows that their description in popular stroke campaigns does not necessarily reflect the experience of patients [59].

In addition to knowledge, self-efficacy and outcome expectation were selected as determinants in developing the matrix of program objectives, even though the survey questionnaires did not specifically explore these constructs. The high proportion of patients referring first to others instead of calling the EMS, as well as available literature evidence, were considered sufficient to support a role for low efficacy as determinant of delay. These psychological constructs were taken into account in developing the message, and emphasis was placed on the need for the patient to feel confident in his/her ability to respond to stroke, as well as in the opportunity of treatment and recovery.

It is noteworthy that, quite unexpectedly, the patient’s decision to first call his/her GP was one of the behavioral covariates related to pre-hospital delay, and that the association persisted in the multivariate analysis. This finding is consistent with one previous report [60] and further supports the need for the educational message to focus on the patient acting promptly without referring to anyone else. It also suggests the need for educational interventions about stroke that specifically target health professionals, most of all general practitioners, who emerged as the source of health information considered most reliable in our context. This is in line with existing evidence, which show that studies that were particularly successful in achieving reductions in prehospital delay adopted a combined multilevel approach to education, incorporating mass media, targeted community education, and professional education [4].

As for the production of program components and materials and the overall design of the campaign, the committee relied on the information obtained by the two surveys to address two aspects: the mode of message delivery and the communication channels.

The narrative mode was selected because of the low levels of education and health literacy observed in the in-hospital sample. Narrative approaches, which include stories, drama, personal experience and the experience of others, are now increasingly employed in health communication, being considered as the basic mode of human interaction and a fundamental way of acquiring knowledge [50]. It is noteworthy that a number of previous stroke campaigns, including the wide national ACT FAST campaigns launched in the UK and in the USA, have used narration to deliver their main message [42–46, 61].

As for the communication channels, in the in-hospital survey, the respondents who correctly attributed symptoms to stroke did not mention as previous sources of information any of those indicated by the community-survey participants. Most of previous information about stroke came through the channel of inter-personal communication, confirming the high grade of social cohesion and community orientation that is typical of our context. For this reason, it was decided to include message dissemination during public events, viewed as an occasion for supplemental access of people to the message, as well as an opportunity to interact face to face with the professionals involved in campaign implementation. Events are considered both a high-impact communication and motivation tool. On the one hand, they are capable of conveying messages extensively and explicitly, maintaining direct contact with the people involved; on the other hand, they can arouse active and participatory interest, which is usually the trigger to change [62]. Nevertheless, events are not often exploited for health campaigns, because of the considerable economic and logistic resources required. We believed this channel would facilitate the dissemination process and message uptake.

The survey results were integrated with evidence from literature and some aspects of the campaign development and planning were mostly defined after a thorough revision of available information on existing campaigns and tools. This approach was used to choose the most suitable appeal, that is the way of organizing the content of the message to make it more likely to persuade or convince people [63]. The stroke campaigns previously implemented in other countries used different appeals, such as the logic/factual (giving facts, figures and information), the emotional or the fear-arousal appeals. Notably, the stroke awareness raising campaign “Act FAST”, which was rolled out in England by the Department of Health between February 2009 and March 2012, used a narrative mode and a fear-arousing appeal, depicting stroke onset as a fire spreading in the brain in a TV advertisement [45, 46]. Therefore, the ‘Act FAST’ campaign mainly focused on displaying stroke as a threat, rather than on the efficacy of the desired response behavior (i.e. calling EMS) and omitted any reference to the effectiveness of thrombolysis. It is noteworthy that the assessment of the effectiveness of the UK campaign according to before-after observational designs gave controversial results, showing in one study [42] a reduction in delay “coinciding” with the start of Act FAST TV campaign that was not confirmed in other reports [64–66]. This supports the concept that fear arousal, i.e. vividly showing people the negative health consequences of life-endangering behaviors, especially when hard-hitting imagery is used, may lead to a defensive reaction, more oriented to avoidance of the fear message than to proper action, when people are not convinced of their self-efficacy or of the effectiveness of the alternative behavior [46, 63]. It was thus agreed to organize the message according to a positive appeal, and to avoid threatening. Indeed, in our storyboard, the patient makes his appearance only after he has been treated and has recovered, to emphasize the efficacy of a correct behavior.

Finally, economic and methodological constraints were taken into account in the design of the overall campaign implementation, especially with regard to the use of mass media. Because of the selection of the stepped wedge cluster randomized design for campaign evaluation (the final step of the intervention mapping framework), the risk of contamination between clusters (the four provinces) was an issue to consider, for the potential overlapping of local media orbits. That is why television public service announcements were not a primary component of our intervention and why the Internet, which was mentioned as a reliable source of health information by the population-survey respondents, could not be included among the dissemination channels.

The choice of the theoretical models for intervention development deserves some further considerations. Many theories were taken into account that could be appropriate to design our educational message [28], but the Andersen Model was deemed as the most suitable to address the issue of delay in patient response as a health threat [38]. Since its publication, it has been used to investigate delay in the diagnosis of many conditions such as myocardial infarction as well as cancer, although it has been employed in different ways in various studies [28]. Some studies were focused on a particular stage, while others attempted to apply the Andersen Model more broadly, with either the aim of identifying the stimulating and impeding factors that influence the transition from one stage to the next, or of determining the length of patient delay stages. In our study, we did not assess the length of the stages but only their contribution to the delay to identify the themes that the educational message should address. In this view, the model has been applied only partially. However, it should be taken into account that our approach had to be adapted to an acute and urgent disease such as stroke, where time intervals between the stages of patient response are not easily measured. Another theoretical model, the Common Sense Model [39] was chosen because it places emphasis on the importance of knowledge and illness representation as determinant of people response to health threats, which was the construct investigated by both surveys.

This study has some limitations. Firstly, the choice to only employ validated instruments in both surveys, rather than developing new tools, prevented us to investigate some aspects that are important for campaign development. Importantly, neither instrument included questions about knowledge of thrombolysis, which would have given some insight into the role of outcome expectation as determinant of the delay in our context. In any case, the questionnaire used by Hsia et al [22] was particularly appropriate for exploring patient behavior according to the General Model of Total Patient Delay. Besides, using validated or published tools enabled us to compare our results with other contexts.

Secondly, because some issues that were taken into account in designing the intervention were not specifically addressed in the Phase 1 surveys, the SC often needed to draw on theory and literature evidence to complete each step of Phase 2, as recommended by the IM framework. Sometimes, the final decision was reached only based on consensus among the SC components, since the available information was not deemed sufficient.

Thirdly, it must be pointed out that the conclusions on which the design of our intervention is based may not be fully generalizable to other populations and countries, because some contextual peculiarities, especially regarding local health organization systems, may vary greatly. It must be pointed out, however, that our primary aim was to design an intervention tailored to our population as much as possible, and that, in any case, the approach we used is generalizable to any other contexts, irrespective of the cultural and social differences.

Fourthly, we acknowledge that the <2 hour cut-off chosen for this study does not fully reflect updated guidelines, which extend criteria for the use of thrombolysis to <3.5/4 hours. This may also imply reduced statistical power of the RCT (where this campaign will be assessed), and limit transfer of findings into practice.

Finally, campaign development would have greatly benefited from the contribution of a sociologist, whose expertise would have enabled to better interpret local barriers and obstacles hindering correct behavior.

In recent years, considerable effort has been made to develop guidelines on reporting complex behavioural interventions. The behaviour change technique taxonomy version 1 [67] is now available and is still under evaluation as a tool for identifying active ingredients within trials of intervention implementation in different fields [68–70] which may help to better inform replication efforts. Unfortunately, this tool was not available at the time our project began, so we could not refer to it for campaign design.

## Conclusions

Following the IM approach, which integrates theories, literature evidence, and data obtained from rigorous context analysis, we developed a communication strategy tailored to our community and, as such, with potential for success. The methodology we followed enabled us to carefully plan the intervention in all its components, and to provide a clear explanation of the reasons that led to key decisions during the intervention development process. This is the prerequisite for adequate monitoring of the different phases of campaign implementation, and will be extremely useful for the evaluation of its effectiveness. On this basis, at the end of the randomized trial that has been planned to evaluate the impact of the campaign, we will also be able to identify the causes of its success or failure.

## Additional file

Additional file 1: Poster of the EROI campaign. (JPG 59 kb)
