# Supplementary figures and images for: Development of an education campaign to reduce delays in pre-hospital response to stroke
Source: BMC Emerg Med. 2017 Jun 24;17:20. doi: 10.1186/s12873-017-0130-9 (PMC5483310; doi:10.1186/s12873-017-0130-9)

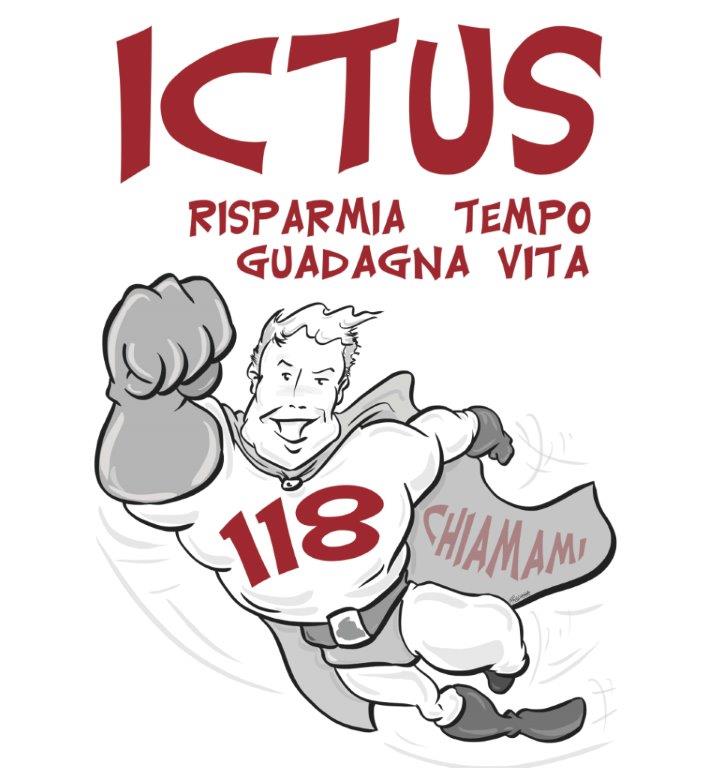

Supplement: Additional file 1: — Poster of the EROI campaign. (JPG 59 kb) [file 12873_2017_130_MOESM1_ESM.jpg]
